# The Impact of Multidimensional Perceived Value on Purchase Intentions for Prepared Dishes in China: The Mediating Role of Behavioral Attitudes and the Moderating Effect of Time Pressure

**DOI:** 10.3390/foods13233778

**Published:** 2024-11-25

**Authors:** Shi Zheng, Leyi Wang, Zhongnan Yu

**Affiliations:** School of Agricultural Economics and Rural Development, Renmin University of China, Beijing 100872, China; zhengshi1974@ruc.edu.cn (S.Z.); yuzhongnan0726@163.com (Z.Y.)

**Keywords:** prepared dishes, willingness to pay, structural equation model, perceived value, time pressure

## Abstract

Accompanied by the flourishing development of China’s prepared dishes industry, understanding consumer purchasing decisions helps companies clarify market needs and expectations. This study analyzed consumers’ willingness to purchase prepared dishes and the moderating role of time pressure using the Theory of Planned Behavior and Structural Equation Modeling. Four hundred and three completed respondents were collected through an online survey of consumers in Beijing, the Capital of China. Our results showed that: (1) Respondents of various demographics (e.g., age, education, occupation, and marital status) had significant differences in willingness to buy. (2) All dimensions of value (functional, emotional, and convenience) had a notable positive effect on behavioral attitudes. However, behavioral attitudes only served as a full mediator between functional value, emotional value, and purchase intentions. (3) Time pressure played a positive moderating role in the effect of perceived value on behavioral attitudes, with positive tendencies in behavioral attitudes increasing as time pressure increased. The study concludes with recommendations for consumers, enterprises, and the government and provides an additional reference for the policy of “Strengthening Food Safety Regulation of Prepared Dishes”.

## 1. Introduction

Prepared dishes (PDs) have become a product of food industrialization by standardizing the production of semi-finished dishes and catering to the needs of the restaurant market to reduce costs and the fast-paced life of consumers [1]. However, due to China’s immature cold chain logistics and food processing technology, the pace of its development has been relatively slow [2]. It was not until the late 1980s that PDs gradually appeared in first-tier cities, such as Beijing and Shanghai [3]. Driven by fast-paced, small-family economics, convenient takeout platforms, and COVID-19, PDs are gaining popularity among the crowd. Data shows that China’s PD market size is rising at a high growth rate of around 20% year-on-year, has reached 516.5 billion yuan in 2023, and is projected to exceed the trillion yuan mark in 2026 [4]. The number of businesses related to PDs has reached a staggering 64,000-plus [5]. In particular, from January to February 2024, the number of newly registered related enterprises reached more than 1320, up 470% compared with the same period in 2022 [4,5]. Meanwhile, a survey by the China Culinary Association shows that the penetration of PDs in China is expected to increase from 15% to 20% by 2030, with a market size of up to 1.2 trillion yuan [6]. Many studies have confirmed that PDs have become a common choice at the consumer table, both offline in superstores and retail outlets, and online on live streaming and e-commerce platforms [7,8,9].

Along with the growth of the industry, consumers are concerned about the quality of PDs. A survey by IMedia Research found that consumers’ concerns about PDs center on unhealthy ingredients and food hygiene, while their purchases are more focused on quality, taste, and ease of cooking [10]. As food safety risks exist in production, processing, storage, transportation, and distribution [11,12], the protection of national policies is particularly important [13]. The “Notice on Strengthening Food Safety Supervision of Prepared Dishes and Promoting High-Quality Development of the Industry” issued by the Chinese government on 18 March 2024 guides and regulates the industry to promote the food safety of PD [14]. Therefore, accurately grasping consumers’ purchasing decision-making processes has become the key to manufacturers’ production.

Most studies have shown that convenience and life stress are the main reasons why consumers purchase PD [15,16]; however, even when there is no time pressure or need to save time, people occasionally purchase PDs to seek new experiences and make life easier [17,18]. For example, a change in a wife’s role from housewife to workplace can prompt families to consume prepared food [19]. Individual heterogeneity also plays a key role in the purchase of PDs. For people who love to cook, the enjoyment of the cooking process is higher, and thus, less likely to consume PDs [20,21,22]. However, for people with fewer cooking skills, consuming PDs becomes their first choice [23]. Consumers who have low trust in food processing, value the naturalness of food, and have more nutritional knowledge consume fewer PDs [24,25], while overweight people have more positive behavioral attitudes toward PDs [26]. Nayga’s study showed that more educated households are more health conscious and, therefore, purchase fewer PDs [27]. However, Park and Capps argued that more educated households have a higher opportunity cost of time and are more likely to purchase PDs [28,29]. Further, the shelf life, origin, taste, and other quality labels of PDs can also have a significant impact on purchasing behavior [29,30]. Thus, the perceived value of PDs varies from person to person, is multidimensional, and is subject to a combination of many factors.

The current research on PDs in China remains at the level of the industry status quo [31,32,33], local cuisine development path [34,35,36], and application technology [37,38,39], while less literature has empirically examined the impact of socio-economic, demographic, and product-related factors on consumers’ willingness to purchase. In terms of consumer acceptance and perception, the unanimous consensus is that trust has a significant positive effect on the continued consumption intention of PDs [40,41] and that positive evaluations among friends can enhance consumers’ favorable perceptions of PDs, thereby increasing purchase intention [42]. Given the unequal information between the two parties in the transaction, selling price and brand are the key bases for assessing the quality and purchase risk of PDs [43]; therefore, a moderate brand premium can have a significant positive effect on consumers’ willingness to purchase [44]. However, high monetary expenditure may reduce consumers’ perception of product value, which in turn weakens their purchase intention [45].

Through comparison, we can find that many types of research on prepared dishes in foreign countries have transitioned from technology, industrial development trends, and consumer purchasing willingness and behavior to nutrient analysis [46]. In China, the differentiation of local cuisine, consumer psychology, and food safety hazards has been discussed [47]. Although some conclusions are different, most of the literature has confirmed that the premise of the development of PDs is food safety, the core competitiveness is convenience, and the industrial pursuit is nutrition [48].

The problem with existing studies is that there is not enough research on the differences in purchase intention and behavior of different prepared foods. Most scholars have studied only the broad categories of convenience foods or prepared foods, ignoring possible differences between products, and to the best of our knowledge, there have been no studies on the perceived value of prepared dishes. In this regard, we propose that prepared foods, especially frozen PDs and prepared net dishes, should be refined to study differences in consumers’ behavioral attitudes, purchase intentions, and behaviors. PDs in this paper refer to semi-finished products that have been processed through moderation and other processes and do not cover prepared net dishes. Usually, PDs need to be stored or transported under cold chain conditions for consumers or processors in the catering chain to simply heat or cook them for consumption. In other words, we refer to PDs as frozen or ambient semi-finished products in supermarkets. The purpose of this study is to investigate the key factors influencing consumers’ purchase of PDs, including (1) The influence of personal characteristics on consumers’ willingness to buy. (2) Whether behavioral attitudes play a mediating role between multidimensional perceived value and purchase intention. (3) Whether time pressure plays a moderating role between perceived value and behavioral attitudes. This study can not only provide targeted marketing suggestions for enterprises but also provide a reference for the government to formulate policies related to PDs.

## 2. Theoretical Framework and Research Hypotheses

### 2.1. Perceived Value Theory and Behavioral Attitudes

Perceived value theory suggests that consumers form a comprehensive evaluation of a product or service by weighing the perceived gains and perceived losses during the purchase process [49]. It is generally recognized that perceived value is multidimensional in nature, but there are different views on the specific division of its dimensions. Babin et al. suggest that perceived value can be divided into utilitarian and hedonic dimensions [50]. Sweeney and Soutar further refined it into multiple dimensions such as quality value, price value, emotional value, and social value [51]. In addition, different categories of products differ in the dimensions of perceived value. The perceived value of disposable goods, durable goods, and luxury goods cannot be generalized [52]. Meanwhile, there are obvious individual differences in consumers’ perceived value of the same product or service. Even for the same person, perceived value may change in different situations and stages of the purchase process [53]. Especially in the online environment, the dimensions of perceived value are more refined, covering a variety of aspects, such as affective value, outcome value, and procedural value. Although a unified standard for the division of dimensions has not yet been formed, the subjective, multidimensional, comparative, and dynamic characteristics of perceived value have been widely recognized [54].

We categorize perceived value into functional value, emotional value, and convenience value for the study. Among these, functional value relates to the ability of PDs to satisfy consumers’ basic needs; emotional value refers to the emotions that consumers experience during the process of purchasing or consuming PDs; and convenience value reflects the fact that PDs satisfy consumers’ needs for short-time cooking. There are two main research approaches to studying the relationship between perceived value and willingness to buy. One is to study the relationship between perceived value and willingness to buy directly [55,56,57,58], and the other is to study the relationship between other variables and willingness to buy by introducing perceived value as a mediating variable to study the mediating effect it plays [59,60,61]. However, it is generally concluded that consumers’ perceived value of products or services is positively related to purchase intention [62]. In addition, perceived value as an important psychological variable is often combined with the Theory of Planned Behavior to study consumer behavior [63,64,65,66].

Accordingly, the following assumptions are proposed:

**H1a:**  *Perceived functional value has a positive effect on consumers’ behavioral attitudes.*

**H1b:**  *Perceived emotional value has a positive effect on consumers’ behavioral attitudes.*

**H1c:**  *Perceived convenience value has a positive influence on consumers’ behavioral attitudes.*

### 2.2. Theory of Planned Behavior and Purchase Intention

The Theory of Planned Behavior is the result of Ajzen’s further development based on the earlier theory of rational behavior [67]. Subjective norms are social pressures that individuals feel about whether to adopt a particular behavior. That is, in predicting the behavior of others, those who have an influence on the individual’s behavioral decision-making group on whether the individual takes a particular behavior to play the size of the influence. Behavioral attitudes refer to the positive or negative feelings an individual has about a behavior. It refers to the attitudes that result from the conceptualization of an individual’s evaluation of that particular behavior. Perceived behavioral control refers to obstacles that reflect an individual’s past experiences and expectations. Perceived behavioral control over behavior is stronger if an individual believes that he has more resources and opportunities at his disposal and expects fewer obstacles.

The Theory of Planned Behavior suggests that individuals may be influenced by both practical and perceived barriers to performing a behavior [67,68]. This theory is regarded as a core theoretical foundation by scholars that explores the influencing factors of behavioral intentions and behavioral prediction. Examples include online shopping [69,70], reproductive decision-making [71], and farmers’ production and marketing behavior [72]. However, the model fails to cover all relevant variables, except for the three basic factors (personal behavioral attitudes, subjective norms, and perceived behavioral control) that influence human behavior. For example, Yan pointed out that there is room for the discovery of new moderating, mediating, and even independent variables beyond basic modules [73]. Nataraajan and Bagozzi further point out that the timing and frequency of an individual’s behavior, as well as past behavior, likewise have a significant effect on intentions and behavior [74].

In the field of food consumption, the Theory of Planned Behavior has been applied in four main ways. The first is the direct use of models to analyze consumption behavioral intentions. Choice consumption behaviors for organic [75], ready-to-eat [76], and meat foods [77] are all discussed. Second, a factor is added to the variables of the base model to analyze consumption behavioral intentions. Olsen et al. [78] introduced ethical behavioral attitudes into the model and found that this factor had a negative effect on people’s intention to consume ready-to-eat foods. Similar research has been conducted on organic food [79] and green food [80]. Among them, in the study on the purchase intention of green vegetables, Zhou added the factors of consumer health, environmental awareness, and consumer perception [80]. Third, the Theory of Planned Behavior is decomposed [81,82]. The fourth is the study of applying a factor of the theory to predict consumer behavioral intention. Some scholars believe that the behavioral attitude variable is influenced by emotional, behavioral attitudes, and moral behavioral attitudes [83], and some studies have shown that information and beliefs through behavioral attitudes affect the consumer’s purchase intention [84].

In this study, behavioral attitudes refer to consumers’ positive or negative evaluations of their purchasing behavior of PDs, reflecting individuals’ psychological tendencies of whether they like PDs. Arvola et al. studied the actual behavior of consumers when purchasing organic food and found that consumers’ willingness to purchase organic food was positively influenced by their attitude [83]. Yin et al. concluded that the conclusions drawn from genetically modified foods were consistent [85]. Zhang’s findings also suggest that willingness to purchase green products is positively influenced by attitude [86]. Subjective norms refer to individuals’ perceived perceptions of their purchasing behavior by friends and family around them. It has been shown that subjective norms can have a positive effect on consumption intentions [87]. Lao also confirmed the facilitating effect of subjective norms on behavioral intention in his study on green consumer behavior [88]. Wang et al. analyzed consumers’ intention to purchase camellia oil and the factors influencing it, and the results also showed that subjective norms have a significant positive effect on purchase intention [89]. Perceived behavioral control is the consumer’s prejudgment of the ease or difficulty of implementing the purchase behavior after evaluating the resources they have and the external environment. For example, whether the purchasing channel is convenient, whether the variety of choices available is sufficient, and whether the financial strength is enough to support the purchasing behavior. Penz et al. discussed consumer behavior when purchasing counterfeit goods, and found that perceived behavioral control had a significant positive effect on purchase intentions [90]. Sheng et al. concluded that perceived behavioral control positively influences consumers’ green purchase intentions [91]. All three will affect the generation of consumers’ willingness to purchase PDs. Time pressure refers to the degree of time sufficiency perceived by an individual. When consumers assess the value of a product as high, higher time pressure may stimulate their behavioral attitudes [16,17]. Djupegot et al. found that ultra-processed foods were consumed more frequently in time-pressured populations, inferring a positive correlation between time pressure and purchasing behavior [92].

Accordingly, the following assumptions are proposed:

**H2:**  *Behavioral attitude has a positive effect on the willingness to purchase PDs.*

**H3:**  *Subjective norms have a positive effect on the willingness to purchase PDs.*

**H4:**  *Perceived behavioral control has a positive effect on the willingness to purchase PDs.*

**H5:**  *Time pressure plays a positive moderating role in the effect of perceived value on behavioral attitudes.*

**Figure 1:**
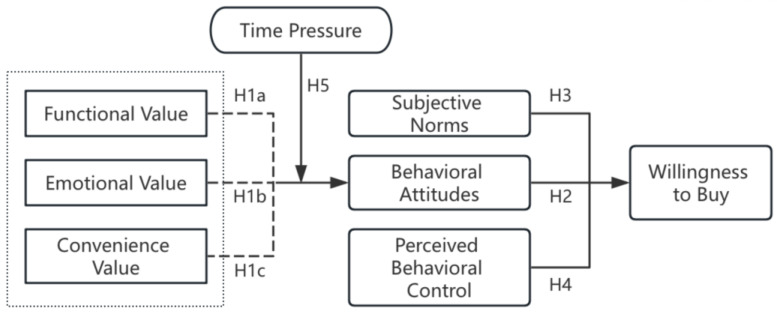
Structural equation model.

In summary, the research framework of this paper is shown in Figure 1.

## 3. Materials and Methods

### 3.1. Data Sources

To ensure the soundness of the study, we first conducted one-on-one face-to-face interviews with 12 PD business leaders and randomly selected consumers between August and October 2023. Four of the business operators were from different PD categories, including meat PD, seafood PD, vegetable PD, and staple PD; the other eight respondents were randomly selected from different age groups. Specific questions in the interviews included basic information about individuals, how much they knew about PDs, their reasons for purchasing PDs, their perceptions of PDs, and their views on current food safety issues. Next, the questionnaire was initially designed and modified based on the interviews. Then, a pre-survey was conducted to ensure the rationality of the questionnaire and validity of the scale. A total of 100 questionnaires were randomly distributed online during the pre-survey, and 84 valid questionnaires were collected.

We found 3 points in our interviews that are worth noting. The first is the lack of a clear perception of the concept of PDs among most of the interviewees. When asked if they had ever purchased one, 4 of them said no. However, it was only when further examples of frozen PDs were given that the interviewees realized that they had had a purchasing experience. Therefore, we clearly listed the definition of frozen PDs and common examples in the questionnaire design. The second is that frozen PDs were not consumed as a premium food item. All interviewees indicated that they never associated the purchase of PDs with social status enhancement. In other words, PDs were not labeled as upscale or luxury by the purchasers but rather as everyday food. Therefore, we believe that some of the perceived value dimensions that apply to green and organic foods, such as the social value dimension, may not be applicable. The third is the diversified motivations for purchasing frozen PDs and the high level of consumer concern about the food safety of PD.

The formal research was based on respondents’ feedback and was conducted from December 2023 to February 2024 using an online approach. The online distribution used a professional third-party questionnaire statistical system, WenJuanXing, to ensure the quality of the random sample data. Among the sample groups of this research are those who live, study, and work in Beijing. A total of 500 questionnaires were distributed and 403 valid questionnaires (80.6%) were collected. The questionnaires with consistent answers for all options (38 respondents), answering time less than 60 s (40 respondents), and some questions with contradictory logic (19 respondents) were screened. It is worth noting that all interviews and questionnaires were approved by the Academic Ethics Committee, and informed consent was obtained from the respondents.

The questionnaire asked about a total of 3 sections. Firstly, the basic information of the individual was investigated, including gender, age, education level, occupation, income, place of residence, and marital status. Secondly, consumers were asked about their purchasing behavior of PDs, including the type and frequency of purchasing PDs. Finally, with reference to Cui and Li’s study [56], a perceived value scale was designed for the characteristics of PDs. Consumers’ perceived value of PDs was categorized into three dimensions: perceived functional value, perceived emotional value, and perceived convenience value (Table 1). Meanwhile, the TPB scale was redesigned with reference to the studies of Han et al. [93], Wang et al. [94], and Lee et al. [95], and the time pressure scale was designed with reference to the study of Brunner et al. [17].

### 3.2. Model

Essentially, consumers’ perceived value, perceived behavioral control, subjective norms, and behavioral attitudes are all part of their psychological activities and cannot be measured directly; therefore, structural equation modeling (SEM) is used to measure them [56,57,60]. Therefore, with the help of AMOS 24.0 software, we verified the effect of perceived value on consumers’ purchases of PD by constructing a structural equation model and using the maximum likelihood method to estimate the fit and path of the model. Considering data quality, theory validation, and causality testing, we used CB-SEM rather than PLS-SEM for our analysis. Further, we refer to the method used by Wang et al. [61] in the moderating effect test to test the moderating role of time pressure in the effect of perceived value on behavioral attitudes.

The following steps were followed to test the moderating effect: first, the model was regressed to determine the correlation coefficients and verify their significance; second, the model was regressed after the interaction, and again, the correlation coefficients were determined and verified for significance. If the coefficient of the interaction term is significant, it proves the existence of a moderating effect and further explores the direction of the moderating effect according to the sign of the coefficient.

## 4. Results

### 4.1. Demographic Characteristics

Women accounted for 53.8% of the participants in the survey, slightly more than men. This proportion is consistent with the fact that women are usually responsible for purchasing food and household goods in most households [45]. In terms of age distribution, young and middle-aged individuals accounted for 93.8% of the sample. Evidently, the age group of 18–55 is the main force of household purchasing and the mainstream consumer group of PDs. It also coincides with the research findings that the convenience of PDs and people’s pursuit of instant food culture drive their consumption behavior [56]. About 76.7% of the respondents have bachelor’s degree or above, more than 70% have stable occupations, with an average monthly household income of more than RMB 3000, and 64% are currently living in the downtown area. This reflects the portrait of this group as highly educated, high-income, stable occupation, and residence. As for marital status, the unmarried population (52.4%) is seven percentage points higher than the married population, which further indicates that the current majority of the single population in first-tier cities is in line with the consumer market for PDs [10]. Table 2 presents the demographic characteristics of the sample.

Similarly, Table 2 demonstrates the impact of demographic variables on willingness to buy. We used an independent samples t-test and one-way ANOVA to explore the differences in consumer purchase decisions across factors such as gender, age, education, occupation, income, place of residence, and marital status. It can be seen that there is no significant difference in consumers’ willingness to buy across gender, income, and place of residence (0.76, 0.39, 0.12, all greater than 0.05), and the significance test results for age, education, occupation, and marital status are less than 0.05, indicating that there is a significant difference in willingness to buy across these dimensions.

It is worth noting that most people only use PDs as supplements to their daily diet. The comparison reveals that 93.3% of consumers have purchased PDs, and occasional purchases are about four times more common than frequent purchases. People tend to choose PDs as an alternative to their daily diet and do not completely rely on them. Meanwhile, Chinese consumers prefer frozen pasta (76.7%), frozen meatballs (68.7%), and semi-finished food (58.1%), and are not keen on frozen dishes that have already been cooked (30.0%). Consumers are not abandoning cooking altogether, but rather reducing the more complex aspects of the cooking process.

### 4.2. Reliability and Validity Analysis

#### 4.2.1. Reliability and Convergent Validity Tests

The results of the reliability and convergent validity tests are shown in Table 3. For the reliability test, we used Cronbach’s alpha coefficient method to ensure the reliability of data quality. Our results show that the Cronbach’s alpha coefficient of each dimension is higher than 0.8, which indicates that the scale has good internal consistency. For the convergent validity test, we referred to the criterion proposed by Fornell and Larcker [96]. The results show that the estimates of standardized factor loadings for each dimension are greater than 0.5, the average variance of refinement (AVE) is greater than 0.5, and the combined reliability (CR) is higher than 0.7, indicating that the dimensions of the scale have good convergent validity.

#### 4.2.2. Exploratory Factor Test

The results of the exploratory factor test are shown in Table 4. The results show that the KMO test coefficient is 0.928, which indicates that the questionnaire possesses good validity.

### 4.3. Normality Test

Referring to the criteria proposed by Kline [97], we tested the skewness coefficient and kurtosis coefficient of the data. Table 5 showed that the maximum absolute value of the skewness coefficient for each question variable dimension was 1.23, which met the criterion of less than three, and the maximum absolute value of the kurtosis coefficient was 1.60, which met the criterion of less than eight. Therefore, it is assumed that the data used meet the criterion of normality and can be subjected to subsequent tests.

### 4.4. Discriminant Validity Test

Discriminant validity tests, as shown in Table 6, were used to test whether the variables of different dimensions were differentiated in terms of content. Our results show that the square root of the AVE for all latent variables exceeds the correlation coefficients between them and the other latent variables, indicating that each latent variable has a high degree of discriminant validity.

### 4.5. Hypothesis Testing

**Figure 2:**
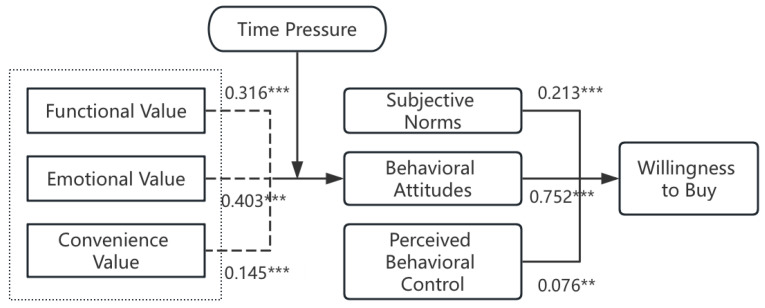
Structural equation modeling results. Note: ** *p* < 0.05; *** *p* < 0.01.

The output of our structural equation modeling diagram using AMOS 24.0 software is shown in Figure 2. The previously proposed hypotheses were tested based on the path coefficient results and their significance. The results show that hypotheses H1a to H4 are valid. Table 7 shows the results of the test of model fitness, which shows that the parameter RMSEA is located in the range of good intervals, and the rest of the parameters are located in the range of excellent intervals. It is evident that the model has good fitness and does not require further corrections.

The path relationship results show that the influence of functional value, emotional value, and convenience value on behavioral attitudes is significantly positive, and hypotheses H1a, H1b, and H1c are valid (Table 8). The strength of influence among the three is in the order of emotional value, functional value, and convenience value. This indicates that the improvement of perceived value can positively promote consumers’ attitudes toward purchasing PD. Behavioral attitudes, perceived behavioral control, and subjective norms have a significantly positive influence on purchase intention, and hypotheses H2, H3, and H4 are valid. The strength of influence among the three is in the order of behavioral attitudes, subjective norms, and perceived behavioral control. This also validates the core findings of the Theory of Planned Behavior.

### 4.6. Mediating Effects Analysis

Considering that behavioral attitudes may play a mediating role in the process of transforming functional value, emotional value, and convenience value into purchase intention [66,98], we analyzed the mediating effect of behavioral attitudes using a bootstrapping mediation effect test. The estimation method for the coefficients is the maximum likelihood method, which sets the number of repeated samples to 5000 and the confidence interval to 90%, i.e., the effect is significant when the upper and lower limits do not contain zero, and P is less than 0.1.

Table 9 showed that the total and indirect effects of functional and affective values on purchase intention were significant, but the direct effect was not significant. Therefore, behavioral attitudes play a fully mediating role between functional value and purchase intention or between emotional value and purchase intention. However, the total effect of convenience value on purchase intention was not significant. Therefore, the mediating effect of behavioral attitudes between convenience value and purchase intention is not considered valid.

### 4.7. Moderating Effects Analysis

To test hypothesis H5, we analyzed the moderating role of time pressure on the effect of perceived value on behavioral attitudes by referring to the method of Wang et al. [61]. The two regression models are shown in Table 10, in which Model 1 did not include an interaction term and mainly analyzed the direct effects of perceived value and time pressure on behavioral attitudes. Model 2, on the other hand, added an interaction term, aiming to reveal how time pressure moderates the effect of perceived value on behavioral attitudes. The results show that the main effect of perceived value on behavioral attitudes is significantly positive, while the coefficient of the interaction term between perceived value and time pressure is 0.087, which passes the test of 0.5% significance. It proves that the presence of time pressure does strengthen the effect of perceived value on behavioral attitudes and plays a positive moderating role; thus, hypothesis H5 is valid.

**Figure 3:**
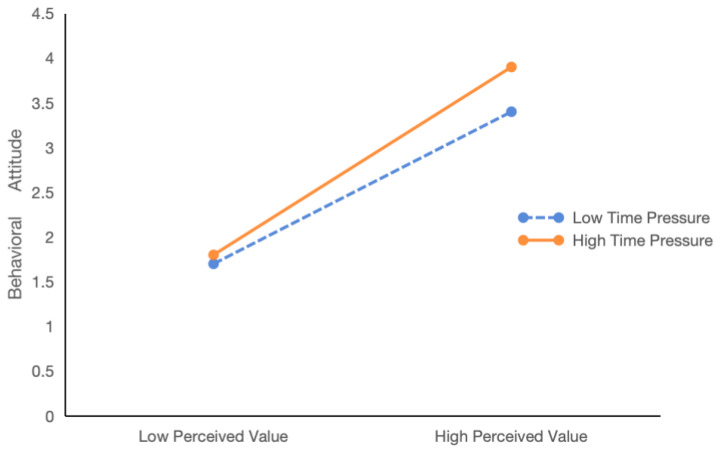
Moderating effects of time pressure.

In addition, in order to show the moderating effect of time pressure more intuitively, we drew a schematic diagram of the moderating effect of time pressure for analysis based on the test results. Figure 3 shows the relationship between time pressure, perceived value, and behavioral attitude. With the strengthening of perceived value, consumers’ behavioral attitudes are strengthened accordingly. Especially in high-time pressure situations, consumers’ behavioral attitudes will show a more obvious enhancement trend compared with low-time pressure situations.

## 5. Discussion and Conclusions

### 5.1. Discussion

Using the Theory of Planned Behavior and the theory of perceived value, this study explores the factors and mechanisms influencing consumers’ purchases of PDs and the moderating role of time pressure from different dimensions of perceived value using behavioral attitudes as the mediating variable. Specifically:

Firstly, perceived value is the basis for consumers’ decision-making in purchasing products and services. In general, the higher the perceived value of a product or service, the more positive the consumer’s behavior. This is consistent with the findings of Zhang et al. [64]. who found that consumers’ perceived value of organic produce positively affects their behavioral attitudes. For PDs, we classified customer-perceived value into three categories. Among them, functional value is reflected in its ability to satisfy consumers’ expectations of consumption [45], emotional value is largely aimed at obtaining post-purchase satisfaction [18], and convenience value is reflected in the fact that consumers save time and energy throughout the process of purchasing and consuming, which enhances the level of personal utility in other aspects [15]. In terms of both functional and emotional value, our view is consistent with that of Sweeney and Soutar [51]. The difference is that, in addition to edible and hedonic consumption motives, we added convenience value to explore and found that convenience value was recognized more than functional and emotional value.

Secondly, the accelerated pace of life and changes in consumption habits have contributed significantly to the acceptance of PDs by consumers, thus gradually diminishing the cost of time spent on cooking. Our view is consistent with the study by Contini et al. [16]. Consumers who face greater time pressure invest relatively less time in home cooking and prefer products that are ready-to-eat or easy to prepare. Unlike in the past, when companies delivered PDs to consumers via the B-side, new retailers such as Freshippo are gradually selling both net dishes and PDs directly to consumers. This move to skip the middleman has also undoubtedly increased consumer accessibility and is in accordance with the consumer image of hungry people rushing into the supermarket to grab a box of PDs to simply cook at home.

Thirdly, behavioral attitudes play different degrees of mediating effects on different dimensions of perceived value. Our results show that the mediating effect of behavioral attitude does not hold between convenience value and purchase intention but plays a fully mediating role between functional value and purchase intention, as well as between emotional value and purchase intention. This means that the edible function of PDs and consumers’ emotions toward them effectively influence purchasing behavior. This may be due to the fact that people’s choice of PDs is influenced by multiple factors such as food safety, taste and flavor, and publicity from friends and family.

The contribution and innovation of this paper is to concentrate on the factors influencing the willingness to purchase frozen PDs and semi-cost category PDs. Considering a more nuanced division, we excluded prepared net dishes from the scope of this paper. First, the extent to which individual consumer factors influence the purchase of PDs was analyzed. Secondly, the convenience value, functional value, and emotional value of PDs were comparatively analyzed, focusing on the convenience value to illustrate the concerns of people’s consumption habits. Further, this study enriches research on PDs in China. Finally, the extent to which behavioral attitudes mediate the different dimensions of perceived value was also explored in depth.

### 5.2. Conclusions

The main conclusions are the following four points:

The convenience value of PDs is recognized more than the functional and emotional values. Frozen dish cuisine, as an emerging category, has not yet been popularized among the population. More than 90% of the population has had experience purchasing PDs, but the vast majority of consumers use PDs as a supplement to their normal diet, and people’s behavioral attitudes and willingness to purchase PDs are generally low. Secondly, consumers of different ages, education levels, occupations, and marital statuses have significant differences in their willingness to purchase PDs. This is not only related to the cooking skills, cooking habits, and time pressure acquired at different ages but also to the opportunity cost of time, which is different for people with different education levels. Housewives have a weaker need to save time and energy than others and, therefore, have significantly weaker purchase intentions than other occupational groups. Unmarried consumers are more willing to buy than married or cohabiting consumers. Thirdly, the higher the perceived value of PDs, the more positive the attitude of consumers toward buying PDs. Consumers’ attitudes toward buying PDs, perceived behavioral control, and subjective norms have a significant positive effect on purchase intention. Meanwhile, behavioral attitudes play a fully mediating role in the effects of functional and emotional values on purchase intention. Finally, time pressure plays a positive moderating role in the influence of perceived value on behavioral attitudes; the stronger the time pressure, the stronger the positive influence of perceived value on behavioral attitudes. Therefore, for people with a faster pace of life, high work pressure, and tighter rest time, less perceived value will cause them to have a stronger behavioral attitude toward PDs, and this group of people is the target customer group of frozen prepared production enterprises.

### 5.3. Limitations and Future Research

We used data from 403 questionnaires to investigate the effects and mechanisms of perceived value on the purchase intention of PDs. Despite providing practical guidance to PD enterprises and the government, there are still shortcomings that can be further studied. The first is the limitation of the research group. Considering the time delay between the release of the national policy and its implementation at the local level, this paper only analyzed the Beijing area. However, the fact that there are regional variations and diverse tastes is a very important factor in the implementation of PDs in China. In the future, we plan to study the differences in perceptions and willingness to purchase PDs between the northern and southern regions and the eastern and western regions. Secondly, we conducted a study based on only three dimensions of the characteristics of PDs (functional value, emotional value, and convenience value). The next step is to refine the division dimensions of perceived value by adding price value, social value, and safety value sections to clarify the attribute weights of consumers’ purchase of PDs. At the same time, PDs are positioned in the research of prepared net dishes in response to China’s Central Document No. 1 of 2023, which reads, “Enhance the level of standardization and normalization of prepared net dishes, central kitchens, and other industries, and cultivate the development of the prepared dish industry”.

The No.1 Central Document is the first policy document issued by the Central Committee of the Communist Party of China and the State Council every year. This policy document is of great significance to China’s agricultural development.

## Figures and Tables

**Table 1:** Research scale.

Variable	Items	Measurement
Functional Value	GN1	Frozen prepared dishes have a great flavor.
	GN2	Frozen prepared dishes are of good quality.
	GN3	Frozen prepared dishes are very nutritious.
Emotional Value	QG1	I don’t worry about food safety when I eat frozen prepared dishes.
	QG2	Frozen prepared dishes make me feel good.
	QG3	I feel comfortable after consuming frozen prepared dishes.
Convenience Value	BL1	Frozen prepared dishes are easy to store.
	BL2	Frozen prepared dishes are easy to cook.
	BL3	Frozen prepared dishes save me time and energy in cooking.
Perceived Behavioral Control	KZ1	Frozen prepared dishes are readily available to me.
	KZ2	Frozen prepared dishes are varied and highly selective.
	KZ3	Frozen prepared dishes are not too expensive, and I can buy them if I want to.
Subjective Norms	GF1	Most people whose opinions I value think I should eat frozen prepared dishes.
	GF2	Most people whose opinions I value would favor me eating frozen prepared dishes.
	GF3	I am surrounded by family and friends who buy frozen prepared dishes on a regular basis.
Behavioral Attitudes	TD1	For me, the act of buying frozen prepared dishes is beneficial.
	TD2	For me, the act of buying frozen prepared dishes is satisfying.
Willingness to Buy	YY1	I will try to buy frozen prepared dishes in the future.
	YY2	I would like to purchase frozen prepared dishes in the future.
	YY3	I intend to purchase frozen prepared dishes in the future.
Time Pressure	SJ1	“So much to do, so little time” is a great quote for me.
	SJ2	I need more time to get my work done.
	SJ3	I feel like no matter how hard I try, I’ll never catch up.

**Table 2:** Descriptive statistics of personal characteristics (N = 403).

Variable	Items	Frequency	Percentage	F	*p*
Gender	Male	186	46.20	0.09	0.76
	Female	217	53.80		
Age	Under 18	5	1.20	5.79	***
	18–25	128	31.80		
	26–40	138	34.20		
	41–55	112	27.80		
	Above 56	20	5.00		
Education	High school or below	94	23.30	4.94	***
	College	40	9.90		
	Undergraduate	122	30.30		
	Graduate or above	147	36.50		
Occupation	Students	115	28.50	2.60	***
	Employees of Government Departments or Institutions	58	14.40		
	Enterprise employees	119	29.50		
	Self-employed	12	3.00		
	Housewife/husband	18	4.50		
	Freelancer	30	7.40		
	Others	51	12.70		
Monthly income (RMB)	≤3000	115	28.50	1.00	0.39
	3000–6000	124	30.80		
	6001–10,000	74	18.40		
	≥10,000	90	22.30		
Residence	Downtown	258	64.00	2.18	0.12
	County	85	21.10		
	Rural	60	14.90		
Marital status	Unmarried	211	52.40	3.40	***
	Married/Cohabiting	183	45.40		
	Divorced/Widowed	9	2.20		
Purchase frequency	Never	27	6.7	—	—
	Rarely	120	29.8		
	Occasionally	203	50.4		
	Frequently	52	12.9		
	Always	1	0.2		
Type of consumption	Frozen semi-finished meats (small crispy meats, chicken cutlets, marinated steaks, etc.)	234	58.1	—	—
	Frozen dishes (pickled fish, boiled pork, fish-flavored meat, etc.)	121	30.0		
	Frozen pasta dishes (dumplings, buns, etc.)	309	76.7		
	Frozen meatballs	277	68.7		

Note: *** *p* < 0.01.

**Table 3:** Reliability and convergent validity test results.

Variable	Items	Estimate	Cronbach’s Alpha	AVE	CR
Functional Value	GN1	0.741	0.820	0.622	0.831
	GN2	0.878			
	GN3	0.739			
Emotional Value	QG1	0.735	0.871	0.712	0.880
	QG2	0.901			
	QG3	0.885			
Convenience Value	BL1	0.857	0.903	0.761	0.905
	BL2	0.892			
	BL3	0.868			
Perceived Behavioral Control	KZ1	0.807	0.863	0.683	0.866
	KZ2	0.867			
	KZ3	0.803			
Subjective Norms	GF1	0.891	0.889	0.736	0.893
	GF2	0.914			
	GF3	0.761			
Behavioral Attitudes	TD1	0.938	0.928	0.866	0.928
	TD2	0.923			
Willingness to Buy	YY1	0.882	0.949	0.865	0.951
	YY2	0.955			
	YY3	0.952			
Time Pressure	SJ1	0.849	0.808	0.594	0.812
	SJ2	0.819			
	SJ3	0.626			

**Table 4:** Exploratory factor test results.

KMO Number of Sample Suitability		0.928
Bartlett’s test of sphericity	Approximate chi-square	7773.542
	Degree of freedom	253
	Significance	0.000

**Table 5:** Normality test results.

Variable	Items	Skewness		Kurtosis	
		Statistics	Standard Error	Statistics	Standard Error
Functional Value	GN1	−0.31	0.12	0.35	0.24
	GN2	−0.27	0.12	0.21	0.24
	GN3	−0.04	0.12	−0.22	0.24
Emotional Value	QG1	0.11	0.12	−0.53	0.24
	QG2	−0.16	0.12	−0.21	0.24
	QG3	−0.05	0.12	−0.02	0.24
Convenience Value	BL1	−1.23	0.12	1.60	0.24
	BL2	−1.08	0.12	0.99	0.24
	BL3	−1.12	0.12	0.89	0.24
Perceived Behavioral Control	KZ1	−0.76	0.12	0.13	0.24
	KZ2	−0.85	0.12	0.43	0.24
	KZ3	−0.53	0.12	0.05	0.24
Subjective Norms	GF1	0.59	0.12	−0.25	0.24
	GF2	0.38	0.12	−0.53	0.24
	GF3	0.06	0.12	−0.61	0.24
Behavioral Attitudes	TD1	−0.30	0.12	−0.51	0.24
	TD2	−0.20	0.12	−0.52	0.24
Willingness to Buy	YY1	−0.08	0.12	−0.43	0.24
	YY2	−0.04	0.12	−0.42	0.24
	YY3	−0.03	0.12	−0.50	0.24
Time Pressure	SJ1	−0.54	0.12	−0.20	0.24
	SJ2	−0.61	0.12	−0.02	0.24
	SJ3	−0.12	0.12	−0.37	0.24

**Table 6:** Discriminant validity test results.

Dimension	FV	EV	CV	PBC	SN	BA	WTB	TP
Functional Value	0.789							
Emotional Value	0.785	0.844						
Convenience Value	0.511	0.423	0.872					
Perceived Behavioral Control	0.618	0.572	0.746	0.826				
Subjective Norms	0.561	0.61	0.176	0.34	0.858			
Behavioral Attitudes	0.731	0.729	0.507	0.632	0.606	0.931		
Willingness to Buy	0.722	0.737	0.431	0.61	0.666	0.909	0.93	
Time Pressure	0.389	0.477	0.494	0.651	0.323	0.478	0.498	0.771

Note: The numbers on the diagonal of the table are the square roots of the mean variance extracted for the corresponding dimensions ($\sqrt{AVE}$ ). The off-diagonal numbers are the inter-dimensional correlation coefficients.

**Table 7:** Structural equation model fitness test.

Parameters	Reference Standard	Actual Results
CMIN/DF	1–3 is excellent, 3–5 is good	2.598
RMSEA	<0.05 is excellent, <0.08 is good	0.063
GFI	>0.9 is excellent, >0.8 is good	0.911
NFI	>0.9 is excellent, >0.8 is good.	0.944
RFI	>0.9 is excellent, >0.8 is good.	0.931
IFI	>0.9 is excellent, >0.8 is good.	0.965
TLI	>0.9 is excellent, 0.8 is good.	0.957
CFI	>0.9 is excellent, 0.8 is good.	0.965

**Table 8:** Model path relationship hypothesis test results.

Path			Standardized Path Coefficient	*p*	Test Results
Functional Value	-->	Behavioral Attitudes	0.361	***	H1a established
Emotional Value	-->	Behavioral Attitudes	0.403	***	H1b established
Convenience Value	-->	Behavioral Attitudes	0.145	***	H1c established
Behavioral Attitudes	-->	Willingness to Buy	0.752	***	H2 established
Perceived Behavioral Control	-->	Willingness to Buy	0.076	**	H4 established
Subjective Norms	-->	Willingness to Buy	0.213	***	H3 established

Note: ** *p* < 0.05; *** *p* < 0.01.

**Table 9:** Mediation effect test results.

	Variable	Estimated Value	Bootstrapping		*p*
			Lower Limit	Upper Bound	
Total effect	Functional Value	0.285	0.131	0.480	***
	Emotional Value	0.340	0.174	0.499	**
	Convenience Value	0.018	−0.099	0.129	0.740
Direct effect	Functional Value	0.024	−0.126	0.171	0.744
	Emotional Value	0.053	−0.063	0.172	0.427
	Convenience Value	−0.098	−0.216	−0.014	*
Indirect effect	Functional Value	0.261	0.108	0.476	**
	Emotional Value	0.287	0.127	0.435	**
	Convenience Value	0.050	0.072	0.188	**

Note: * *p* < 0.1; ** *p* < 0.05; *** *p* < 0.01.

**Table 10:** Moderating effect test results.

Variable	Model 1		Model 2	
	Coefficient	Standard Error	Coefficient	Standard Error
Gender	−0.035	0.071	−0.026	0.070
Age	−0.065	0.057	−0.058	0.056
Education	−0.002	0.050	−0.005	0.049
Occupation	0.027	0.024	0.024	0.023
Monthly income(RMB)	0.012	0.036	0.010	0.035
Residence	0.050	0.058	0.049	0.058
Marital status	0.019	0.095	0.004	0.095
Perceived Value	0.888 ***	0.055	0.900 ***	0.055
Time Pressure	0.164 ***	0.042	0.190 ***	0.043
Perceived Value * Time Pressure			0.087 **	0.039
$R^{2}$	0.529		0.535	
Adjustment $R^{2}$	0.519		0.523	
F	49.100 ***		45.109 ***	

Note: * *p* < 0.1; ** *p* < 0.05; *** *p* < 0.01.

## Data Availability

The data that support the findings of this study are available from the corresponding authors upon reasonable request. The data are not publicly available because of privacy restrictions.

## References

[1] Li Y., Li H., Mi X., Dong S., Wang C., Pan S. (2024). Current Situation and Development Countermeasures of Prepared Dishes Industry in China. China Fruit Veg..

[2] Feng J. (2024). Current Situation, Problems and Countermeasure Suggestions for the Development of Prepared Dishes in China. China Food Ind..

[3] Wang D., Huang Y., Lu M. (2023). Current Status of Prepared Food Research. Liaoning Agric. Sci..

[4] People’s Daily Online Research Institute Prepared Dishes Industry Development Report. http://yjy.people.com.cn/n1/2023/0710/c440911-40031856.html.

[5] SkyEye Big Data Report (2021). How to celebrate New Year’s Eve locally. China Econ. Wkly..

[6] Ma J., Qiao Q., Zhao F., Luo L., Hao L. (2024). China’s Prepared Dishes Industry Status and Development Proposals. Storage Process..

[7] Wang Y. (2022). Opportunities of Cold Chain Logistics in the Hot Market of Premade Cuisine. Logist. Mater. Handl..

[8] Wu X., Rao L., Zhang H., Hu X., Liao X. (2022). Quality and Safety Improvement of Premade Cuisine by Novel Food Processing Technologies. J. Food Sci. Technol..

[9] Yi B., Xu H. (2023). Research and Development Status of Prepared Foods in China: A Review. Appl. Sci..

[10] Media Research A New Chapter in Flavored Foods: Ai Media’s 2024 Market Research Report on China’s Flavored Foods Industry Shockingly Released. https://baijiahao.baidu.com/s?id=1798448953528091300&wfr=spider&for=pc.

[11] Zhang L., Zhang C. (2024). The Risk of Food Safety in Prefabricated Dishes Industry and Its Countermeasures. Sci. Technol. Cereals Oils Foods.

[12] Zhang Y., Chen X., Fan C., Wang J. (2023). Research on Related Standards and Risk Management of ‘Prepared Foods’. Chin. J. Food Hyg..

[13] Yan X., Guo L. (2024). Research on Food Safety Supervision Dilemma and Legal Regulation of Prepared Dishes. Storage Process..

[14] Food Production Safety Supervision and Management Department Notice on Strengthening Food Safety Supervision of Prepared Dishes and Promoting High-Quality Development of the Industry. https://www.gov.cn/zhengce/zhengceku/202403/content_6940808.htm.

[15] Tharrey M., Drogue S., Privet L., Perignon M., Dubois C., Darmon N. (2020). Industrially Processed Iv Home-Prepared Dishes: What Economic Benefit for the Consumer?. Public Health Nutr..

[16] Contini C., Boncinelli F., Gerini F., Scozzafava G., Casini L. (2018). Investigating the Role of Personal and Context-related Factors in Convenience Foods Consumption. Appetite.

[17] Brunner T.A., van der Horst K., Siegrist M. (2010). Convenience Food Products. Drivers for Consumption. Appetite.

[18] Botonaki A., Mattas K. (2010). Revealing the Values Behind Convenience Food Consumption. Appetite.

[19] Redman B.J. (1980). The Impact of Women’s Time Allocation on Expenditure for Meals Away from Home and Prepared Foods. Am. J. Agric. Econ..

[20] Raimundo L.M.B., Batalha M.O., Sans P. (2020). Consumer Attitudes Towards Convenience Food Usage: Exploring the Case of São Paulo, Brazil. J. Int. Food Agribus. Mark..

[21] Costa A.I.A., Schoolmeester D., Dekker M., Jongen W.M.F. (2007). To Cook or Not to Cook: A Means-end Study of Motives for Choice of Meal Solutions. Food Qual. Prefer..

[22] Candel M. (2001). Consumers’ Convenience Orientation Towards Meal Preparation: Conceptualization and Measurement. Appetite.

[23] Hartmann C., Dohle S., Siegrist M. (2013). Importance of Cooking Skills for Balanced Food Choices. Appetite.

[24] Drescher L.S., de Jonge J., Goddard E., Herzfeld T. (2012). Consumer’s Stated Trust in the Food Industry and Meat Purchases. Agric. Hum. Values.

[25] Botonaki A., Natos D., Mattas K. (2008). Exploring Convenience Food Consumption Through a Structural Equation Model. J. Food Prod. Mark..

[26] Van der Horst K., Brunner T.A., Siegrist M. (2011). Ready-meal Consumption: Associations with Weight Status and Cooking Skills. Public Health Nutr..

[27] Nayga R.M. (1998). A Sample Selection Model for Prepared Food Expenditures. Appl. Econ..

[28] Park J.L., Capps O. (1997). Demand for Prepared Meals by US Households. Am. J. Agric. Econ..

[29] Stranieri S., Ricci E.C., Banterle A. (2017). Convenience Food with Environmentally-Sustainable Attributes: A Consumer Perspective. Appetite.

[30] Ahlgren M.K., Gustafsson I.-B., Hall G. (2005). The Impact of the Meal Situation on the Consumption of Ready Meals. Int. J. Consum. Stud..

[31] Wang W., Zhang R., Zhang J., Wu Z. (2022). Status Quo, Problems and Future Prospects of Prepared Dishes. Meat Res..

[32] Wang J., Gao Q., Lou W. (2023). Development Status and Trends of the Pre-prepared Food Industry in China. Mod. Food Sci. Technol..

[33] Zhao C., Chen S., Li W., Yang G., Niu W., Hao Z., Zhang L. (2023). Analysis of Issues in the Development of the Pre-prepared Dishes Sector. Mod. Food Sci. Technol..

[34] Wang J., Liu M., Chen Y., Deng N., Zhang B., Li C., Xiao Z., Fang F., Liu D., Yang D. (2022). Analysis of Current Situation and Development Path of Prepared Dishes Industry in Hunan. J. Chin. Inst. Food Sci. Technol..

[35] Xu Y., Zhang Y., Zhou F., Chen S. (2022). Analysis on the Development Mode and Current Situation of Prepared Dishes in Guangdong. J. Chin. Inst. Food Sci. Technol..

[36] Zhang Y., Chen H. (2022). Analysis on the Current Situation and Development Path of Prefabricated Dishes Industry Derived from Sichuan Cuisines. J. Chin. Inst. Food Sci. Technol..

[37] Huang H., Chen S., Zhao Y., Cen J., Yang S., Wang Y., Xiang H., Li L., Yang X., Wu Y. (2022). Research Advances on Processing and Quality Safety Control Technology of Aquatic Pre-made Products. South China Fish. Sci..

[38] Wang J., Ren G., Hou Z., Xiong J. (2020). Analysis of Microbial Contamination Status and Influencing Factors in Pre-cooked Food Enterprises. Chin. J. Food Hyg..

[39] Shan D., Yang H., Xie L., Wei Y. (2023). Application of Sterilization Technology in Improving the Quality and Safety of Prepared Food. Packag. Eng..

[40] Luo F., Shang W. (2023). Pre-Made Meal Products Information Defects Lead to Product Crisis and Consumer Mental Injury. Econ. Manag..

[41] Zhang W., Zheng J., Li Y. (2024). Explaining Chinese Consumers’ Continuous Consumption Intention toward Prepared Dishes: The Role of Perceived Risk and Trust. Foods.

[42] Fu Y., Zhang W., Wang R., Zheng J. (2024). How Cognition Influences Chinese Residents’ Continuous Purchasing Intention of Prepared Dishes under the Distributed Cognitive Perspective. Foods.

[43] Wang L. (2005). On the Effect of Product External Cues on Consumer Purchase Intention under Information Asymmetry. Consum. Econ..

[44] Zhang C., Lu J. (2014). Research on the Influential Factors of Agricultural Product Regional Brand Purchase Intention. Soft Sci..

[45] Feng J., Mu W., Fu Z. (2006). A Review of Research on Consumers’ Willingness to Buy. Mod. Manag. Sci..

[46] Bognár A., Piekarski J. (2000). Guidelines for Recipe Information and Calculation of Nutrient Composition of Prepared Foods (Dishes). J. Food Compos. Anal..

[47] Du S., Li M., Yang F., Zhou J. (2024). A Structural Equation Model-Based Study on Factors Influencing Consumers’ Intention to Purchase Pre-made Dishes. China J. Commer..

[48] Wang S., Zhu H., Dong H., Zheng Y., Qu C. (2023). The Development Status and Prospect Analysis of Pre-made Dishes. Farm Prod. Process..

[49] Zeithaml V.A. (1988). Consumer Perceptions of Price, Quality, and Value—A Means-end Model and Synthesis of Evidence. J. Mark..

[50] Babin B.J., Darden W.R., Griffin M. (1994). Work and/or fun: Measuring Hedonic and Utilitarian Shopping Value. J. Consum. Res..

[51] Sweeney J.C., Soutar G.N. (2001). Consumer Perceived Value: The Development of A Multiple Item Scale. J. Retail..

[52] Chen J., Wang F. (2012). Effect of Perceived Values on Consumer Purchase Intention among Commodity Category. J. Syst. Manag..

[53] Dong D., Yang Y. (2008). Theoretic Analysis of Perceived Value by Consumers under Internet Environment. Chin. J. Manag..

[54] Fan X., Luo H. (2003). Study on Competitiveness of Service Firms: A Customer Perceived Value Perspective. Nankai Bus. Rev..

[55] Shi H. (2015). Research on Urban Residents’ Willingness for Low-carbon Consumption based on Logistic Model. J. Beijing Inst. Technol. (Soc. Sci. Ed.).

[56] Cui D., Li S. (2018). The Influence of Customer Perceived Value of Featured Agricultural Products on Customer Purchase Behavior—Based on Multi-group Structural Equation Model. J. Agrotech. Econ..

[57] Li G., Yang P., Zheng S. (2019). The Impact of Ingredient Brand Perceived Value on Consumers’ Repurchase Intention—An Empirical Study with Brand Trust as the Mediator. Mod. Manag..

[58] Liu Y., Wang Y. (2021). Perceived Value, Satisfaction and Behavioral Intention of Consumer Featured Agricultural Products: Based on Donkey Meat Consumption of Urban Residents in Shandong and Hebei Provinces. J. China Agric. Univ..

[59] Liu H. (2015). The Impact of Regulatory Focus and Communication Strategy on Purchase Intention: The Mediating Effect of Perceived Value. Tour. Trib..

[60] Quan C., Fan Y. (2018). An Empirical Study on the Impact of Logistics Service Quality and Customer Satisfaction under Cross-border Online Shopping—The Mediating Role of Perceived Value. J. Harbin Univ. Commer. (Soc. Sci. Ed.).

[61] Wang J., Wang L., Wang M. (2019). How Ewom and Perceived Value Influence Purchase Intention: An Investigation of Mediation-Moderation Effects. J. Ind. Eng. Eng. Manag..

[62] Wang J., Li J. (2021). Research on Consumers’ Cognition, Emotion and Willingness to Safety Certified Agricultural Products under Information Asymmetry—Empirical Study Based on 12 Cities in East China. World Agric..

[63] Xue Y., Bai X., Hu Y. (2016). An Empirical Study on Perceived Value and Anticipated Regret Affecting Intention to Purchase Green Food. Soft Sci..

[64] Zhang G., Yang L., Xu Z. (2020). Influence of Customers’Perceived Value on Their Willingness to Purchase Organic Agricultural Products. Guangdong Agric. Sci..

[65] Wang J., Gao Z. (2020). An Analysis of Consumers’ Purchasing Path of Safety-certified Pork Based on Behavioral Characteristics and Situational Factors: A Micro Survey from 12 Cities in East China. Chin. Rural Econ..

[66] Xu X., Liu Y. (2024). Formation Mechanism of the Purchase Intention of Green Agricultural Products from the Perspective of Consumers’ Perception: Based on the Expanded Model of Reasoned Action Theory. J. China Agric. Univ..

[67] Ajzen I. (1991). The Theory of Planned Behavior. Organ. Behav. Hum. Decis. Process..

[68] Fishbein M., Ajzen I. (1977). Belief, Attitude, Intention, and Behavior: An Introduction to Theory and Research. Philos. Rhetor..

[69] Lim H., Dubinsky A.J. (2005). The Theory of Planned Behavior in E-commerce: Making a Case for Interdependencies Between Salient Beliefs. Psychol. Mark..

[70] Zhang H., Bai C., Li C. (2011). Study on Consumers’ Online Purchasing Intention—Comparison between TRA and TPB. Soft Sci..

[71] Mao Z., Luo H. (2013). Difference Between Fertility Intention and Fertility Behavior for Women Subject to the Two-children Policy: An Empirical Study Based on the Theory of Planned Behavior. Popul. Res..

[72] Shi H., Wang Z., Yan L. (2019). The Influence of Ecological Cognition on Farmers’ Grain for Green Behavior: Based on TPB and Multi-group SEM. China Land Sci..

[73] Yan Y. (2014). A Review on the Origins and Development of the Theory of Planned Behavior. Chin. J. Journal. Commun..

[74] Nataraajan R., Bagozzi R.P. (1999). The Year 2000: Looking back. Psychol. Mark..

[75] Chen M. (2007). Consumer Attitudes and Purchase Intentions in Relation to Organic Foods in Taiwan: Moderating Effects of Food-related Personality Traits. Food Qual. Prefer..

[76] Mahon D., Cowan C., McCarthy M. (2006). The Role of Attitudes, Subjective Norm, Perceived Control and Habit in the Consumption of Ready Meals and Takeaways in Great Britain. Food Qual. Prefer..

[77] Seffen A.E., Dohle S. (2023). What Motivates German Consumers to Reduce Their Meat Consumption? Identifying Relevant Beliefs. Appetite.

[78] Olsen N.V., Sijtsema S.J., Hall G. (2010). Predicting Consumers’ Intention to Consume Ready-to-eat Meals. The Role of Moral Attitude. Appetite.

[79] Robinson R., Smith C. (2002). Psychosocial and Demographic Variables Associated with Consumer Intention to Purchase Sustainably Produced Foods as Defined by the Midwest Food Alliance. J. Nutr. Educ. Behav..

[80] Zhou H., Wang D. (2019). Study on Purchase Intention of Green Vegetable Consumers Based on Structural Equation Model. North. Hortic..

[81] Contini C., Boncinelli F., Marone E., Scozzafava G., Casini L. (2020). Drivers of Plant-based Convenience Foods Consumption: Results of A Multicomponent Extension of the Theory of Planned Behaviour. Food Qual. Prefer..

[82] Qi X., Ploeger A. (2019). Explaining Consumers’ Intentions Towards Purchasing Green Food in Qingdao, China: The Amendment and Extension of the Theory of Planned Behavior. Appetite.

[83] Arvola A., Vassallo M., Dean M., Lampila P., Saba A., Lähteenmäki L., Shepherd R. (2008). Predicting Intentions to Purchase Organic Food: The Role of Affective and Moral Attitudes in the Theory of Planned Behaviour. Appetite.

[84] Luo C. (2010). Analysis of Factors Influencing Consumers’ Willingness to Pay for Safe Food- Based on Theoretical Framework of Planned Behavior. China Rural Surv..

[85] Yin Z., Cheng P., Yuan X., Wang Y. (2012). Formation of Consumers’ Willingness to Purchase Genetically Modified Foods: Theoretical Modeling and Empirical Tests. Consum. Econ..

[86] Zhang D. (2021). Research on Consumers’ Consumption Behavior of Green Products from the Perspective of Environmental Concern. Price Theory Pract..

[87] Tarkiainen A., Sundqvist S. (2005). Subjective Norms, Attitudes and Intentions of Finnish Consumers in Buying Organic Food. Br. Food J..

[88] Lao K. (2013). Research on Mechanism of Consumer Innovativeness Influences Green Consumption Behavior. Nankai Bus. Rev..

[89] Wang Z., Weng N., Liu W. (2015). Research on the Willingness to Buy Camellia Oil of Consumer: Based on Survey Data of Consumer in Fujian Province. For. Econ..

[90] Penz E., Stoettinger B. (2004). Forget the “Real” Thing—Take the Copy! An Exploratory Model for the Volitional Purchase of Counterfeit Products. Adv. Consum. Res..

[91] Sheng G., Gong S., Xie F. (2019). Theoretical Basis and Empirical Test of the Formation of Chinese Consumers’Green Purchasing Intention. Jilin Univ. J. Soc. Sci. Ed..

[92] Djupegot I.L., Nenseth C.B., Bere E., Bjørnarå H.B.T., Helland S.H., Øverby N.C., Torstveit M.K., Stea T.H. (2017). The Association Between Time Scarcity, Sociodemographic Correlates and Consumption of Ultra-Processed Foods Among Parents in Norway: A Cross-Sectional Study. BMC Public Health.

[93] Han H., Hsu L.-T., Sheu C. (2010). Application of the Theory of Planned Behavior to Green Hotel Choice: Testing the Effect of Environmental Friendly Activities. Tour. Manag..

[94] Wang Y., Wiegerinck V., Krikke H., Zhang H. (2013). Understanding the Purchase Intention Towards Remanufactured Product in Closed-loop Supply Chains An empirical study in China. Int. J. Phys. Distrib. Logist. Manag..

[95] Lee J.-S., Hsu L.-T., Han H., Kim Y. (2010). Understanding How Consumers View Green Hotels: How A Hotel’s Green Image Can Influence Behavioural Intentions. J. Sustain. Tour..

[96] Fornell C., Larcker D.F. (1981). Evaluating Structural Equation Models with Unobservable Variables and Measurement Error. J. Mark. Res..

[97] Kline R.B. (1998). Software Review: Software Programs for Structural Equation Modeling: Amos, EQS, and LISREL. J. Psychoeduc. Assess..

[98] Wang Y., Huang L. (2019). Research on the Impact of Mobile Short Video Perceived Value on Consumers’ Purchase Intention. Econ. Manag..

